# Attachment in Old Age: A Fundamental Pillar for Public Health and Wellbeing

**DOI:** 10.3389/ijph.2025.1607770

**Published:** 2025-05-21

**Authors:** Carol Rodway

**Affiliations:** Maimónides University, Buenos Aires, Argentina

**Keywords:** attachment, aging, older adult, healthy aging, public health

**The IJPH series “Young Researcher Editorial” is a training project of the Swiss School of Public Health.**

Given that attachment is a process influencing the entire life cycle, its consideration in healthcare should not be restricted solely to childhood. In the face of the biopsychosocial challenges of aging, this editorial proposes integrating Attachment Theory (AT) into public health policies, particularly within Primary Healthcare (PHC), as a key strategy to enhance the quality, continuity, and effectiveness of care for older adults. The article presents theoretical foundations, recent empirical evidence, and intervention proposals aimed at incorporating this perspective into clinical and community practice.

Attachment Theory, developed by John Bowlby (1969), posits that early emotional bonds shape how individuals interact and regulate emotions throughout life. These bonds form Internal Working Models (IWMs), which are especially reactivated during periods of vulnerability, such as old age [1].

With the global aging of the population, challenges related to mental health, multimorbidity, and social isolation have intensified. In this context, attachment emerges as a key social determinant of wellbeing and continuity of care [2].

During old age, attachment relationships adapt to new contexts. Secure relationships, particularly with healthcare providers, offer a protective buffer against adversity. Recent literature highlights that secure attachment in older adults improves treatment adherence, strengthens emotional regulation, and fosters trust in medical care [3].

Nevertheless, attachment in older adults is rarely addressed and is poorly represented in current public health policies [4]. Strategies such as Person-Centered Care (PCC), self-care promotion, or empowerment address relational dimensions, but do not explicitly integrate AT. This omission may limit the effectiveness of such interventions by neglecting the emotional needs of older adults [5, 6].

For older adults, relationships with health professionals are crucial, as they often face multimorbidity and mental health challenges that demand trust and support from families, caregivers, society, and especially the healthcare team. Secure attachment fosters trust, enhances adherence to medical advice, facilitates disease management, and provides emotional stability [2]. Integrating AT would acknowledge that the quality of the therapeutic bond directly influences care outcomes. Furthermore, it would contribute to transcending autonomy-focused approaches by incorporating interdependence as a core axis of healthy aging [7].

Promoting secure attachment in old age requires interventions that transform and complement current practices in PHC, aligned with public health approaches in the development, implementation, evaluation, and scaling of evidence-based interventions to improve and sustain individual and population health [8].

We propose three concrete lines of action as a complementary framework for public policy in PHC:

- (a) Training healthcare personnel: Educating teams to recognize attachment styles and their impact on therapeutic relationships and health outcomes. This includes active listening, emotional regulation, and empathic communication skills [3].
- (b) Redesigning the clinical model: Creating care strategies that incorporate the patient’s relational history, including attachment assessments and open dialogue strategies to enhance continuity and trust [9].
- (c) Psychosocial programs based on AT: Implementing group interventions that address attachment insecurities and strengthen peer bonds to improve emotional health and reduce isolation [10].

These strategies should be implemented through specialized professionals in AT, interdisciplinary teams, and community-based programs that ensure a continuous and systematic emotional care approach.

Moreover, addressing attachment should be considered a shared responsibility among governments, healthcare teams, academic institutions, community organizations, and families. This requires [5]:

- Policies that finance intergenerational programs.
- Curricular reforms to include AT in professional training.
- Promotion of emotionally safe environments.

Only coordinated action will ensure dignified, healthy, and emotionally sustained aging.

Integrating Attachment Theory into the care of older adults through PHC enhances clinical effectiveness, fosters more human therapeutic relationships, and optimizes wellbeing in later life. Incorporating this framework into public policies strengthens the equity, sustainability, and adaptive capacity of health systems. AT, as both a clinical and organizational tool, enables a cultural shift toward relationship-centered care and upholds the relational dignity of aging.

## Data Availability

The original contributions presented in the study are included in the article/supplementary material, further inquiries can be directed to the corresponding author.
