# Erratum: Attachment in Old Age: A Fundamental Pillar for Public Health and Wellbeing

**DOI:** 10.3389/ijph.2025.1609270

**Published:** 2025-11-19

**Authors:** Swiss School of Public Health Production Office

**Affiliations:** Frontiers Media SA, Lausanne, Switzerland

**Keywords:** attachment, aging, older adult, healthy aging, public health

Due to a production error, Rosario Spencer was included as an author in the published article. This inclusion was not approved by the journal.

The publisher apologizes for this mistake.

The original version of this article has been updated.

